# Variant calling: Considerations, practices, and developments

**DOI:** 10.1002/humu.24311

**Published:** 2021-12-16

**Authors:** Stepanka Zverinova, Victor Guryev

**Affiliations:** ^1^ European Research Institute for the Biology of Ageing University of Groningen, University Medical Centre Groningen Groningen The Netherlands

**Keywords:** best practices, genome sequencing, method development, variant calling

## Abstract

The success of many clinical, association, or population genetics studies critically relies on properly performed variant calling step. The variety of modern genomics protocols, techniques, and platforms makes our choices of methods and algorithms difficult and there is no “one size fits all” solution for study design and data analysis. In this review, we discuss considerations that need to be taken into account while designing the study and preparing for the experiments. We outline the variety of variant types that can be detected using sequencing approaches and highlight some specific requirements and basic principles of their detection. Finally, we cover interesting developments that enable variant calling for a broad range of applications in the genomics field. We conclude by discussing technological and algorithmic advances that have the potential to change the ways of calling DNA variants in the nearest future.

## Graphical abstract

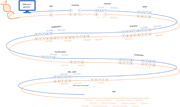

## 1 INTRODUCTION

Genome and exome sequencing techniques are rapidly replacing genotyping microarrays in diagnostic and prognostic studies. A key advantage of new technologies is their ability to give a nearly complete view on the content of our genomes with digital precision. With recent improvements in scalability and constantly decreasing prices, genome sequencing has all potential for being a method of choice for a broad range of genomics studies. However, as a relatively new invention that also has multiple technological peculiarities, the analysis of genome variants using sequencing data is less standardized compared to genotyping arrays. In this review, we will discuss current approaches to variant calling using sequencing data and specifically focus on considerations while planning genomics experiments, practices while executing them, and promising developments to consider for future projects.

## 2 CONSIDERATIONS

DNA sequencing is popular among researchers and companies, one cannot complain about the limited choice of platforms and kits for preparing and sequencing the samples. Before engaging in a genomics project using sequencing, it is a good idea to consider different options and choose the one that matches your research question and provides the most cost‐efficient solution.

### 2.1 DNA isolation and fragmentation

The basic experimental procedures such as DNA isolation can have systematic effects on the representation of different genomic regions (van Heesch et al., 2013) and, as a consequence, on variant detection.

Also, DNA fragmentation, a necessary step for short‐read library preparation, can be achieved through a variety of methods, such as mechanical fragmentation, sonication, acoustic and hydrodynamic shearing, chemical or enzymatic fragmentation. The most recent addition to the portfolio of fragmentation methods is Tn5 transposases that can “deliver” polymerase chain reaction (PCR) or sequencing adaptors directly to DNA (Picelli et al., 2014). Choices on DNA sources, isolation and fragmentation methods can vary from project to project, but it is a good idea to agree on these standards before starting a collaborative project involving multiple research groups. The quality and quantity of genomic DNA determine the need for PCR amplification and can greatly affect the quality of variant calls.

### 2.2 PCR duplicates

Redundant reads originating from the same DNA fragment introduced during the PCR amplification of a library are common artifacts created during genome sequencing. Such artifacts can falsely increase allele frequency or even introduce erroneous mutation calls. There are several ways to either remove or mark the PCR duplicates to lower the false‐positive calls. When sufficient input material is available, modern protocols allow for amplification‐free library construction. When the input material is scarce and does not meet requirements for PCR‐free library preparations, the individual molecules can be barcoded by adaptors with unique molecular identifiers (UMIs). Even after the PCR amplification of such a library, UMIs will help to discard PCR duplicates. When neither PCR‐free prep nor UMIs were used, it is a common practice to mark additional reads with shared mapping coordinates as potential duplicates. However, this computational method might overcorrect (duplicated regions) or miss (repetitive regions) the real duplicates.

### 2.3 Genome coverage

Choice of sequencing strategy has an important effect on the average depth of the genome coverage. Short‐read whole‐genome sequencing offers the most complete approach and typically yields 30×, while long reads with lower per‐base quality are routinely sequenced to 60× coverage. Targeted resequencing, that involves selection of genomic regions, exhibits a rather nonuniform coverage of target regions (e.g., whole‐exome sequencing). As sufficient coverage is a key parameter in variant calling, exome sequencing employs an even higher 90×–100× average coverage to compensate for uneven coverage among selected regions. Interestingly, some projects deliberately turn to a lower sequencing depth, when sequencing related individuals or looking for common association signals in a large population (CONVERGE Consortium, 2015; Genome of the Netherlands Consortium, 2014).

### 2.4 Platform and read length

**Figure 1:**
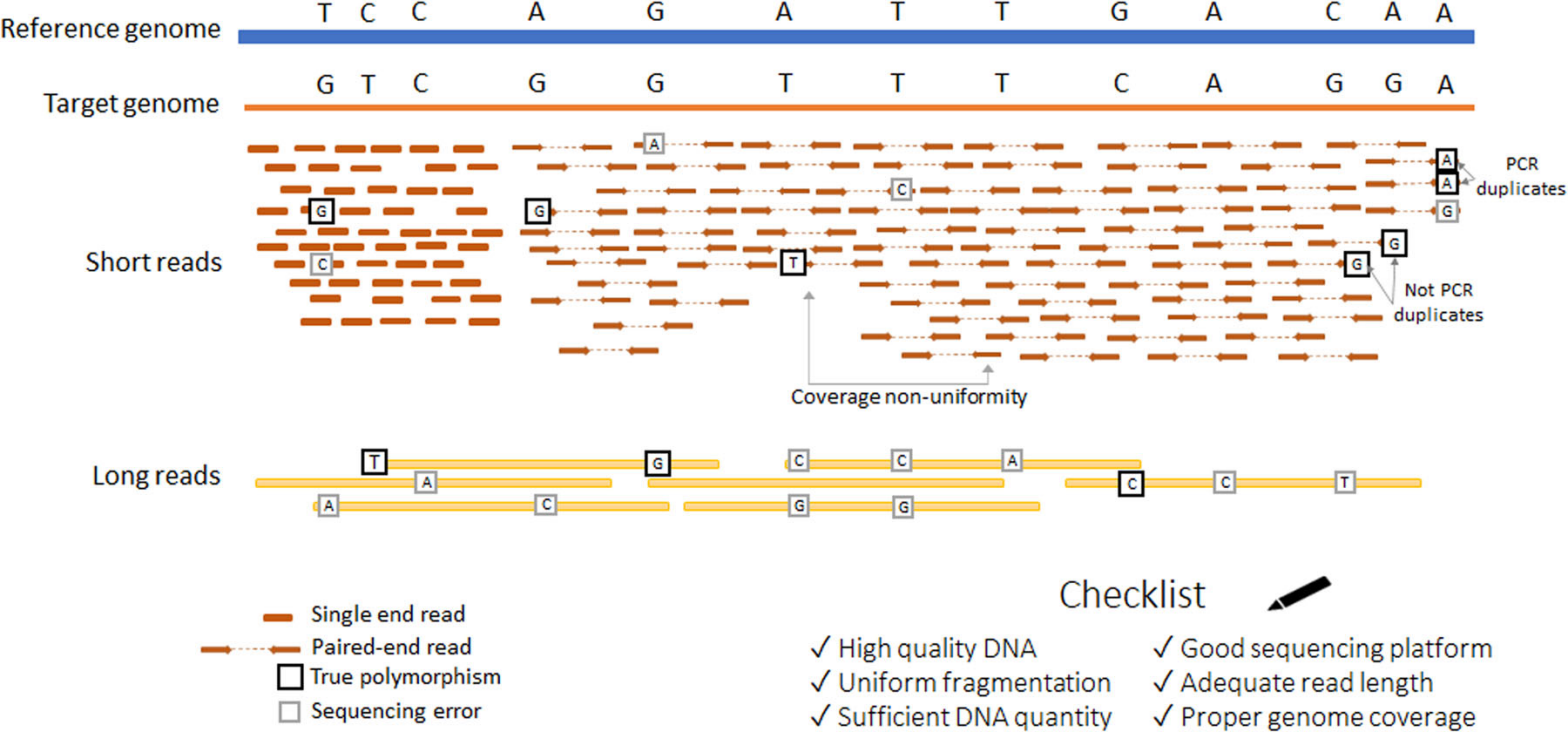
Overview of experimental factors that are important for planning and performing a genome sequencing study

Short reads sequencing (typical reads span several hundred bases) is currently the most popular and cost‐effective strategy for variant detection. As price is typically proportional to the number of bases read, the preference should be given to the longest possible reads (available for this platform) and paired‐end over single‐end mode (Figure 1). This will allow to uniquely address a bigger proportion of the genome, improve detection of structural variants and improve the performance of read assembly methods. Overall, short read‐based methods are well‐established, cheap, have low error rates, but can fail to distinguish recently duplicated, repetitive sequences and can under‐represent DNA segments with very high or low GC content. On the other hand, long reads can cover tens and hundreds of kilobases, which is advantageous for calling of larger structural variants as they can span over large repeats and regions with GC content bias. However, the costs per base, as well as error rates, are higher than those for short reads. Several interesting alternatives to obtain high‐quality data with perfect mapping ability of long reads rely on advanced barcoding of short reads (linked‐reads), circular consensus reading (PacBio high fidelity reads), and combining long‐ and short‐read sequencing data (Wenger et al., 2019; Zhang et al., 2020).

To conclude, we tried to outline some of the important choices that need to be made at the beginning and might affect the efficiency of calling and completeness of the resulting variant set. When your experience with variant calling is rather limited it is recommended to involve a data analysis specialist at the stage of project conception rather than after data is produced. This can help to avoid issues of lacking power or poor ability to call certain types of variants from one side and wasting the resources from another.

## 3 PRACTICES

### 3.1 Choice of genome reference

A poor choice of the reference assembly version can affect the results and thus should be carefully considered beforehand based on the aim of the study. Inclusion of alternative haplotypes for hypervariable regions such as major histocompatibility locus (MHC), pseudoautosomal regions of chromosome Y may result in loss of unique mapping for some of the genes and, hence, reduced sensitivity of variant calling. On the other hand, the inclusion of unplaced and unlocalized contigs will prevent the mismapping of reads originating from these sequences and prevent many false‐positive calls. It is a general recommendation to use the so‐called primary assembly version (chromosomes, mtDNA, unplaced and unlocalized contigs) of the reference genome unless a specific aim of the study calls for the use of an extended version.

### 3.2 Aligning

To avoid the multiple sources of errors in data, such as amplification biases, errors introduced during sequencing and base calling, and mapping artifacts that arise during reads alignment, the data must be appropriately pre‐processed. At this stage, it is preferable to prioritize sensitivity over specificity, to ensure the abundance of potential variants, rather than missing any. A good example of balanced preprocessing steps is described in Genome Analysis ToolKit (GATK) best practices tutorial (DePristo et al., 2011; GATK_Team, 2021), and can be used as a guide to constructing a workflow according to the established and validated analysis principles. The pipeline uses a BWA‐MEM aligner, a robust mapping algorithm, to map the sequence data to a reference genome (H. Li, 2013). An optional next step involves marking PCR duplicates for PCR‐amplified libraries, for example using SAMtools package (H. Li et al., 2009) or according to manufacturer recommendation if UMIs were employed. GATK is a set of tools that can be easily used to perform the rest of preprocessing and variant calling routines. A common practice in data preprocessing is base quality score recalibration (BQSR). Base quality is a confidence score provided by the sequencer for each DNA base in the data and often needs recalibration to correct for any systematic bias, which can arise during library preparation and genome sequencing. The recalibration process uses covariate measurements from the base call in the data set to build and apply an adjustment model, resulting in highly accurate base quality scores.

### 3.3 Calling small variants

The most common type of changes observed in our genomes is a single nucleotide variant (SNV), a substitution of a single base at a certain position in the genome. A typical genome sequencing project detects several million SNVs per individual human sample. When reads are properly aligned to the genome reference, calling a standalone single‐base “typo” is rather straightforward. Apart from the GATK program suite mentioned above, several others such as BCFtools and FreeBayes are popular tools that can generate a list of small variants in a computation‐efficient way (Table 1) (Danecek et al., 2021; Garrison & Marth, 2012). Interestingly, summary characteristics of SNVs can serve as quality control parameters in population sequencing studies. Thus, a biased transition to transversion ratio can indicate errors in SNV analysis or a presence of strong mutational bias in the genome.

**Table 1:** Selected list of tools commonly used for detection of DNA variants

Tool	Approach, method	Application	References
Small variants			
GATK Haplotypecaller	Local reassembly of haplotypes	Germline, MNPs	(Poplin, Ruano‐Rubio, et al., 2018)
BCFtools	Positional, pileups	Germline	(Danecek et al., 2021)
FreeBayes	Haplotype‐based, Bayesian model	Germline, MNPs	(Garrison & Marth, 2012)
GATK Mutect2	Local reassembly	Somatic	(Cibulskis et al., 2013)
Strelka2	Tiered haplotype model	Germline, somatic	(Kim et al., 2018)
Structural variants			
Delly2	RP, SR, RD	Germline SVs	(Rausch et al., 2012)
Pindel	SR, RP	Germline SVs	(Ye et al., 2018)
Manta	SR, RP, AS	Germline, somatic	(Chen et al., 2016)
GRIDSS2	AS, SV Breakpoint	Somatic	(Cameron et al., 2021)
Varscan2	RD, Circular Binary Segmentation	Exome, somatic, CNVs	(Koboldt et al., 2012)
EXCAVATOR2	RD, In‐,Off‐target	Exome, CNVs	(D'Aurizio et al., 2016)
ExomeDepth	RD, beta‐binomial	Exome, CNVs	(Plagnol et al., 2012)
Other, exotic variants			
Mobster	RP, clipped reads	MEIs	(Thung et al., 2014)
Expansion‐Hunter	Reads spanning, flanking, in‐repeat	Repeat expansion	(Dolzhenko et al., 2017)
sideRETRO	SR, RP at insert	GRIP	(Miller et al., 2021)
Harpak et al. (2017)	HMM model	NAGC	(Harpak et al., 2017)
Li et al. (2019)	k‐mer count, MDS	NUMT	(W. Li et al., 2019)

Abbreviations: AS, assembly; CNV, copy‐number variants; GATK, Genome Analysis ToolKit; MEI, mobile element insertions; RD, read depth; RP, read pairing; SR, split‐read; SV, structural variants.

Other small variants that lead to small insertion or deletion between 1 and 20 DNA bases are commonly known as indels. These changes are shorter than a sequencing read and can be found by realigning partially mapped or unmapped reads using split‐read aligners (Ye et al., 2018).

The biggest challenges in calling small variants present themselves in hypervariable and repetitive regions of the genome. Both tightly clustered variants and look‐a‐like regions in the genome present problems for small variant calling. The former can be alleviated by haplotype‐aware calling where, instead of “walking” genome one position at a time, polymorphic regions are located and their sequences are assembled from local sequencing reads, resulting in longer stretches of allelic sequences that can be mapped more accurately. The latter problem can be resolved by using longer sequencing reads that overlap a sufficient number of bases that are different between otherwise very similar regions of the genome.

### 3.4 SV calling

**Figure 2:**
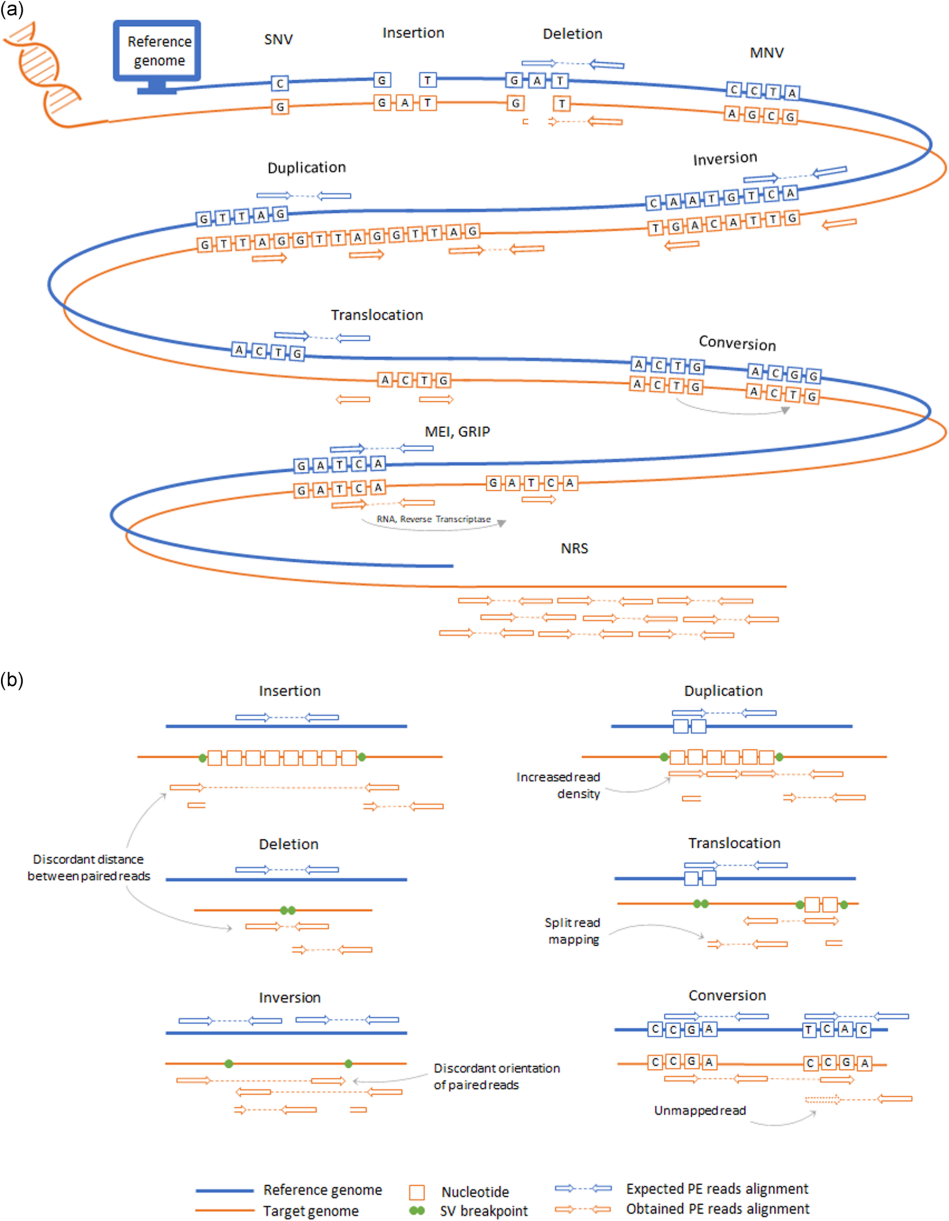
Diversity of DNA variant types. (a) Variants that can be discovered by comparing reference genomes and mapped NGS reads. (b) Identification of structural variants using signatures from mapped read pairs. MEI, mobile element insertions; MNV, multi‐nucleotide variants; NRS, non‐reference sequences; SNV, single nucleotide variants; SV, structural variants

Larger variants that encompass 20 and more DNA bases, also known as structural variants (SVs), are difficult to locate from individual short reads. Alterations that encompass multiple base pairs typically interfere with the mapping of reads that overlap this type of DNA variants (Figure 2a). These aberrant mapping signatures indicate structural differences between reference and patient genome and can be used for the detection of structural variants (Figure 2b). The following types of signatures have been proven as informative for SV discovery:

(i) Change in read density. Large deletions and duplications result in segmental increase or decrease of DNA copy number and, as a result, contribute to a local change of read density, when compared to unaffected regions. These two types of SVs, collectively known as copy‐number variants (CNVs) can be identified by segmentation of read coverage profiles using algorithms involving hidden Markov models (HMM) or circular binary segmentation (CBS).

(ii) Altered distance between paired reads. When the distance between mapping positions of paired reads significantly deviates from the expected size of the DNA fragment, it may indicate local DNA content difference between reference and genome of the patient. A longer distance between mapped paired reads indicates more DNA bases in reference than in the patient sample and hence deletion, while a shorter distance can be a hallmark of an insertion.

(iii) Discordant orientation of paired reads. When aligned, paired reads typically map to opposite DNA strands. In rare cases when one read from a pair overlaps the breakpoint of an SV, the relative mapping of reads in a pair can exhibit unexpected directionality. Thus, if one of the reads ends in an inverted segment, both reads would map to the same DNA strand. Similarly, tandem duplications frequently show inverse read order (upstream read on reverse, downstream read on forward strand) since, in the patient DNA, these reads belong to two sequential copies of the same reference DNA segment.

(iv) Unmapped reads. When an alignment of a read to the reference genome fails, it might indicate that the read overlaps a breakpoint of a structural variant. In this case, split‐mapping of this read may reveal the large variants. With many possibilities of arbitrary splitting of an unmapped short read, it is difficult to avoid mis‐mapping. However, the genomic coordinate of the mapped “mate” read from the same pair can be used as a proxy to find the start of the SV breakpoint.

The state‐of‐the‐art SV discovery combines these aforementioned SV signatures in a clever way. Thus, a relative position of read pairs allows discrimination between tandem and dispersed CNVs, which is impossible from the read density analysis alone. The robust and specific SV calls, therefore, rely on multiple independent read pairs and are supported by multiple SV signatures (Figure 2b).

In some of the applications, the assumptions underlying SV signatures might be violated. For example, read density in exome data is very nonuniform and CNV detection methods need to discriminate between effects of change in copy number and technical variability in fragmentation and target selection efficiency. This is especially challenging for small sample sets and single‐exon CNVs (Gordeeva et al., 2021).

It is worth noting that read position/orientation and especially read depth signatures cannot resolve an SV breakpoint at base‐pair resolution. In this situation, split‐read mapping and local de novo assembly help with fine‐mapping of the SV coordinates.

### 3.5 Other and exotic variant types

Some of the variant types need special approaches for their discovery and were frequently missed by previous generations of variant calling software. We will briefly discuss their origins and the specifics of their identification.

Some mutational mechanisms, like defective polymerase zeta (Harris & Nielsen, 2014) can lead to a replacement of multiple bases before a displacement of the faulty enzyme. Such multi‐base variants, known as multi‐nucleotide variants (MNVs) or complex indels will be inherited as one allele, but can be called as several independent SNVs/indels if the variant caller (e.g., GATK Unified Genotyper) operated in “one base at a time mode.” A “split” reporting of an MNV can affect the prediction of its functional effect, as individual base changes at codon positions can have different effects on amino acid sequence when interpreted separately. Haplotype aware calling, (e.g., GATK Haplotype Caller) alleviates this problem and should correctly represent this variant type.

Even a non‐faulty polymerase can make errors when it encounters certain types of DNA sequences. Mononucleotide and polynucleotide tandem repeats are good examples of such challenging regions. Polymerase slippage and incorrect reannealing of DNA strands can lead to expansion or contraction of such repeats. Expectedly, the longer the repeat, the more unstable it will behave during DNA replication. Longer repeats of this kind have been previously implicated in multiple genetic disorders such as fragile X syndrome or amyotrophic lateral sclerosis (Swinnen et al., 2020). Allelic variants with such repeats, especially high‐risk alleles, frequently exceed read and even fragment size typical in short‐read sequencing, making their identification challenging. Several software packages were developed to estimate the length of such repeats from genome sequencing data (Bahlo et al., 2018).

Next to tandem repeats, there are also dispersed repeats appearing in our genomes. A common type of dispersed repeats is mobile elements (MEs)—“parasitic” DNA sequences with the ability to copy and/or propagate themselves across DNA. Autonomous elements, such as long interspersed nuclear element 1 (LINE‐1) encode for enzymes that can convert its RNA to DNA and insert an extra copy of itself into the genome. Nonautonomous elements are typically short (e.g., SINEs) and use enzymes produced by their autonomous “cousins” for their propagation. Nearly half of our genome may in fact be the result of the past activity of mobile elements. The arms race between these “egoistic” DNA elements and cellular processes suppressing their replication limits the number of functional full‐size copies around a hundred LINEs per human genome (Penzkofer et al., 2017). The sheer presence of ME‐derived sequences in genomes makes identification of their new insertions challenging, especially so when a new copy “lands” inside another repeat. Several software packages utilize read pair, split‐read, and local assembly to locate and genotype mobile element insertions (MEI) in WGS data (Chu et al., 2020; Gardner et al., 2017; Thung et al., 2014; Torene et al., 2020).

The presence of reverse transcription (RNA‐to‐DNA) functionality opens a possibility for other RNA molecules to “enter” our genomes. In rare cases, a regular processed human mRNA can be recognized by enzymes encoded by LINE‐1, resulting in the introduction of retrocopy of a gene. Such gene retrotransposition insertion polymorphisms (GRIPs) typically represent intronless, partial, and nonfunctional copies of actual genes (Ewing et al., 2013). They can obscure variant discovery of the original gene when GRIP is rare in the population and is not part of genome reference or standard variant set. Regular variant calling software would typically call GRIPs as duplications and/or transpositions, but specialized solutions exist to locate and genotype them properly (Miller et al., 2021).

Apart from mobile elements and retrocopies of genes, nuclear genomes can contain (partial) inserts of mtDNA. These nuclear inserts of mitochondrial DNA (termed NUMTs) can be detected using discordantly mapped read pairs or alignment‐free methods (W. Li et al., 2019) and can be applied to the whole genome or exome data when the target list includes mitochondrial genes.

The presence of recently inserted copies of mobile elements, (retro‐) copies of genes, and mtDNA segments that are not represented in the genome reference introduces additional challenges in variant calling. The alignment of paralogous sequences to the same segment of genome reference leads to the addition of paralogous sequence variants to the list of true polymorphisms. This problem can be alleviated by extending the reference to incorporating new inserts (see section “Completeness of genome reference” below).

Another genetic mechanism that can result in large‐scale changes in our DNA is non‐allelic gene conversion (NAGC)—a consequence of improper DNA repair event when a highly similar, but not identical copy of segment is used as a template during homologous recombination repair (Harpak et al., 2017). This mechanism tends to eliminate differences between recently duplicated regions, but can also result in loss of function events when a functional copy of a gene is rewritten using its pseudogene as a template. Such events seem to be rare, difficult to detect from short sequencing reads, and, at a first sight, can look like a set of multiple independent polymorphisms.

Many of the challenges in SV discovery mentioned above are caused by limitations of short‐read sequencing and will be solved through democratizing long‐read sequencing, with most of SVs being detected from long reads alignments (Miga et al., 2020).

## 4 DEVELOPMENTS

### 4.1 Improvement of variant calling methods

While the first generation of variant callers typically relied on a single algorithmic approach, the latest software solutions combine multiple signatures (such as read depth, partially and discordantly mapped reads) and methods (split‐read mappings, gapped alignment, de novo assembly) for the identification of complex and structural variants (Rausch et al., 2012). However, as there is still no single tool that has mastered all methods and variant types, consolidation of variant call sets from multiple tools has become a popular approach (Zarate et al., 2020).

**Figure 3:**
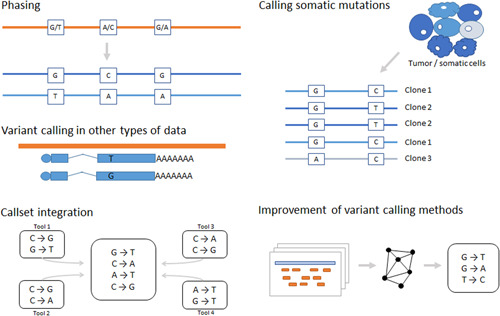
Current developments and challenges in variant identification technologies and algorithms

Next to this synthesis, the new methods are constantly added to our toolbox (Figure 3). With the increasing popularity of new deep‐learning approaches, such as convolutional neural networks, computers can generate pileup image representations and call variants from them. An implementation of such a deep‐learning algorithm can call germline SNVs and indels and essentially requires just a single parameter defining the minimum variant quality threshold (Poplin, Chang, et al., 2018).

### 4.2 Variant calling for other data types

Due to the popularity of sequencing methods, they are also frequently employed for profiling transcriptomes, epigenomes, regulatory proteins attached to DNA or RNA, and so on. These data types can have several important differences that need to be taken into account when calling variants.

Similar to exome sequencing, other data types do not necessarily have a uniform coverage throughout the whole genome, so that one cannot rely on the completeness and or same detection power for the variants called from such data.

Alongside, data set‐specific peculiarities need special attention by variant calling software. A good example is gapped alignment common for splicing events in RNA‐seq data. It is important to prevent misalignment cases due to assigning small trailing parts of the read to an intron. Thus, transcriptome aligners have functionality for two‐stage mapping: identifying all novel splice junctions first (preferably for all samples in the study) and then using them in the second mapping stage to avoid misalignments and false SNPs (Dobin et al., 2013). Further, gaps in the alignment may complicate the variant discovery process, hence some software solutions such as GATK split exonic blocks into multiple supplementary alignments to simplify variant calling.

Another peculiarity of non‐genomic data is that some of the observed variants do not represent DNA state, but are introduced by technical or biological modifications. Bisulfite conversion during methylation profiling and RNA editing are good examples of such processes. Although modifications of these types have a very clear nucleotide context and substitution type, separating them and real DNA variants with the same signature can be difficult (Barturen et al., 2013; Lo Giudice et al., 2020).

Last but not least, most of the data types do not necessarily show an equal representation of both alleles. Differences in allele expression or haplotype‐specific protein binding can bias the allelic ratio from the 50%–50% expected in genome or exome sequencing and therefore the separation of true variants and sequencing errors becomes more difficult. In this case, it can be a good idea to perform validation of variant discovery, for example by comparing results against independent genotyping, such as SNP arrays or checking of calls' consistency across family members.

### 4.3 Calling somatic mutations

Similar challenges are being faced when research centers around calling variants in cancer or somatic cells. Tumor heterogeneity, the presence of both tumor and nontumor cells, and clonal expansions lead to a broad distribution of mutated allele frequencies. As a result, very different, more lenient settings are used for calling somatic compared to germline variants and sometimes even different software solutions that are developed specifically for somatic variant calling (e.g., GATK Mutect2 and other tools listed in Table 1) (Cibulskis et al., 2013).

When using archived samples, the storing regime may play a crucial role in data quality and interpretation. While high‐quality DNA can be readily extracted from research‐grade fresh frozen samples, the majority of specimens available for researchers are stored as formalin‐fixed paraffin‐embedded (FFPE) samples. The latter show multiple signs of DNA deterioration including degradation, DNA cross‐linking, fragmentation, strand breaks, base deamination, and abasic sites. To boost the specificity of variant calling for FFPE samples, the processing of multiple biopsies per patient can be a good strategy. For example, paired tumor and control (DNA from nontumor tissue, blood, or saliva) experiment design improves the interpretation of tumor sequencing results and aids in differentiating between somatic and germline mutations.

The ability to call somatic changes holds great prospects for early diagnostics of human diseases. The recent advances in sample preparation and sequencing technologies enable the profiling of cell‐free DNA circulating in the blood, allowing for real‐time, noninvasive profiling of cancer or monitoring disease treatment (Herberts & Wyatt, 2021).

### 4.4 Phasing variants

In some cases, it is not sufficient to know genotypes to evaluate their involvement in disease. If multiple potentially deleterious alleles exist in a gene, it is important to understand if a single or both copies of a gene are affected, which requires phasing of alleles into haplotypes (Tourdot et al., 2021). Although short‐range haplotype information is processed and reported by some of the variant callers, gene‐ and chromosome‐level phasing often needs extra efforts and can be obtained through information about read pairing (physical phasing), genomes of close relatives, and phased haplotypes from population sequencing projects (computational phasing) and even new experimental methods (Porubsky et al., 2017).

### 4.5 Completeness of genome reference

The non‐reference sequences (NRS) represent genomic segments detected in sequencing data, which are currently not represented in the human genome reference. These sequences are often polymorphic in the population and thus can be considered as a type of structural variation (Hehir‐Kwa et al., 2016; Naslavsky et al., 2020). The NRS may contain new complete or parts of genes as well as noncoding DNA sequences potentially influencing transcription. Integration of data from various genome sequencing projects will help to identify, catalog, map and characterize these segments from the whole human population and build a pangenome reference for the unbiased interpretation of genomics data.

### 4.6 Long reads technologies

Third‐generation sequencing technologies (e.g., developed by Pacific Biosciences and Oxford Nanopore) offer numerous advantages over short‐read sequencing. Their main strength lies in the ability to routinely produce reads that are tens and hundreds of thousands of kilobases long. Such long reads simplify de novo assembly, improve the unambiguity of read mapping, and the detection of structural variants. Long‐read platforms are also able to provide insight into the nature and frequency of nucleotide modifications, such as methylation. Nevertheless, long‐read sequencing is still more costly than short‐reads technology and can be expensive for large sequencing projects. Further, their relatively high base error rate usually requires consensus polishing of high coverage data or combining of long‐read and short‐read data to achieve maximum base call accuracy. The recent and remarkable advances in long‐read technologies, such as high fidelity (HiFi) reads, provide proof of principle that these technologies are instrumental to detect the most challenging variants, such as mid‐sized SVs on repetitive sequence background, and decode end‐to‐end representation of human chromosomes (Miga et al., 2020). The resulting completion of human chromosome X demonstrated the possibility to sequence the genome with only a few remaining gaps and the need for further development of methods for the analysis of segmental duplications and satellite arrays.

## 5 CONCLUSIONS

In this paper we reviewed the common practices of variant calling, focusing on preparatory steps, decisions to be made, diversity of variant types, and basic principles of their identification. As the pace of technological developments in genome sequencing does not seem to slow down, we can expect that many obstacles we encounter today will disappear. As a result, researchers would be able to obtain a comprehensive set of variants more easily and dedicate more of their time to clinical and biological questions.

## References

[humu24311-bib-0001] Bahlo, M. , Bennett, M. F. , Degorski, P. , Tankard, R. M. , Delatycki, M. B. , & Lockhart, P. J. (2018). Recent advances in the detection of repeat expansions with short‐read next‐generation sequencing. F1000Research, 7, 736. 10.12688/f1000research.13980.1 PMC6008857 29946432

[humu24311-bib-0002] Barturen, G. , Rueda, A. , Oliver, J. L. , & Hackenberg, M. (2013). MethylExtract: High‐quality methylation maps and SNV calling from whole genome bisulfite sequencing data. F1000Research, 2, 217. 10.12688/f1000research.2-217.v2 24627790 PMC3938178

[humu24311-bib-0003] Cameron, D. L. , Baber, J. , Shale, C. , Valle‐Inclan, J. E. , Besselink, N. , van Hoeck, A. , Janssen, R. , Cuppen, E. , Priestley, P. , & Papenfuss, A. T. (2021). GRIDSS2: Comprehensive characterisation of somatic structural variation using single breakend variants and structural variant phasing. Genome Biology, 22(1), 202. 10.1186/s13059-021-02423-x 34253237 PMC8274009

[humu24311-bib-0004] Chen, X. , Schulz‐Trieglaff, O. , Shaw, R. , Barnes, B. , Schlesinger, F. , Källberg, M. , Cox, A. J. , Kruglyak, S. , & Saunders, C. T. (2016). Manta: Rapid detection of structural variants and indels for germline and cancer sequencing applications. Bioinformatics, 32(8), 1220–1222. 10.1093/bioinformatics/btv710 26647377

[humu24311-bib-0005] Chu, C. , Zhao, B. , Park, P. J. , & Lee, E. A. (2020). Identification and genotyping of transposable element insertions from genome sequencing data. Current Protocols in Human Genetics, 107(1), e102. 10.1002/cphg.102 32662945 PMC8906366

[humu24311-bib-0006] Cibulskis, K. , Lawrence, M. S. , Carter, S. L. , Sivachenko, A. , Jaffe, D. , Sougnez, C. , Gabriel, S. , Meyerson, M. , Lander, E. S. , & Getz, G. (2013). Sensitive detection of somatic point mutations in impure and heterogeneous cancer samples. Nature Biotechnology, 31(3), 213–219. 10.1038/nbt.2514 PMC3833702 23396013

[humu24311-bib-0007] CONVERGE Consortium . (2015). Sparse whole‐genome sequencing identifies two loci for major depressive disorder. Nature, 523(7562), 588–591. 10.1038/nature14659 26176920 PMC4522619

[humu24311-bib-0008] Danecek, P. , Bonfield, J. K. , Liddle, J. , Marshall, J. , Ohan, V. , Pollard, M. O. , Whitwham, A. , Keane, T. , McCarthy, S. A. , Davies, R. M. , & Li, H. (2021). Twelve years of SAMtools and BCFtools. GigaScience, 10(2), giab008. 10.1093/gigascience/giab008 33590861 PMC7931819

[humu24311-bib-0009] D'Aurizio, R. , Pippucci, T. , Tattini, L. , Giusti, B. , Pellegrini, M. , & Magi, A. (2016). Enhanced copy number variants detection from whole‐exome sequencing data using EXCAVATOR2. Nucleic Acids Research, 44(20), 154. 10.1093/nar/gkw695 PMC5175347 27507884

[humu24311-bib-0010] DePristo, M. A. , Banks, E. , Poplin, R. , Garimella, K. V. , Maguire, J. R. , Hartl, C. , Philippakis, A. A. , del Angel, G. , Rivas, M. A. , Hanna, M. , McKenna, A. , Fennell, T. J. , Kernytsky, A. M. , Sivachenko, A. Y. , Cibulskis, K. , Gabriel, S. B. , Altshuler, D. , & Daly, M. J. (2011). A framework for variation discovery and genotyping using next‐generation DNA sequencing data. Nature Genetics, 43(5), 491–498. 10.1038/ng.806 21478889 PMC3083463

[humu24311-bib-0011] Dobin, A. , Davis, C. A. , Schlesinger, F. , Drenkow, J. , Zaleski, C. , Jha, S. , Batut, P. , Chaisson, M. , & Gingeras, T. R. (2013). STAR: ultrafast universal RNA‐seq aligner. Bioinformatics (Oxford, England), 29(1), 15–21. 10.1093/bioinformatics/bts635 PMC3530905 23104886

[humu24311-bib-0012] Dolzhenko, E. , van Vugt, J. , Shaw, R. J. , Bekritsky, M. A. , van Blitterswijk, M. , Narzisi, G. , Ajay, S. S. , Rajan, V. , Lajoie, B. R. , Johnson, N. H. , Kingsbury, Z. , Humphray, S. J. , Schellevis, R. D. , Brands, W. J. , Baker, M. , Rademakers, R. , Kooyman, M. , Tazelaar, G. , van Es, M. A. , … Eberle, M. A. (2017). Detection of long repeat expansions from PCR‐free whole‐genome sequence data. Genome Research, 27(11), 1895–1903. 10.1101/gr.225672.117 28887402 PMC5668946

[humu24311-bib-0013] Ewing, A. D. , Ballinger, T. J. , Earl, D. , Broad Institute Genome Sequencing and Analysis Program and, P. , Harris, C. C. , Ding, L. , Wilson, R. K. , & Haussler, D. (2013). Retrotransposition of gene transcripts leads to structural variation in mammalian genomes. Genome Biology, 14(3), R22. 10.1186/gb-2013-14-3-r22 23497673 PMC3663115

[humu24311-bib-0014] Gardner, E. J. , Lam, V. K. , Harris, D. N. , Chuang, N. T. , Scott, E. C. , Pittard, W. S. , Mills, R. E. , Genomes Project Consortium , & Devine, S. E. (2017). The Mobile Element Locator Tool (MELT): Population‐scale mobile element discovery and biology. Genome Research, 27(11), 1916–1929. 10.1101/gr.218032.116 28855259 PMC5668948

[humu24311-bib-0015] Garrison, E. , & Marth, G. (2012). Haplotype‐based variant detection from short‐read sequencing. http://arxiv.org/abs/1207.3907

[humu24311-bib-0016] GATK_Team . (2021). GATK best practices. https://gatk.broadinstitute.org/hc/en-us/articles/360035535912-Data-pre-processing-for-variant-discovery

[humu24311-bib-0017] Genome of the Netherlands Consortium . (2014). Whole‐genome sequence variation, population structure and demographic history of the Dutch population. Nature Genetics, 46(8), 818–825. 10.1038/ng.3021 24974849

[humu24311-bib-0018] Gordeeva, V. , Sharova, E. , Babalyan, K. , Sultanov, R. , Govorun, V. M. , & Arapidi, G. (2021). Benchmarking germline CNV calling tools from exome sequencing data. Scientific Reports, 11(1), 14416. 10.1038/s41598-021-93878-2 34257369 PMC8277855

[humu24311-bib-0019] Harpak, A. , Lan, X. , Gao, Z. , & Pritchard, J. K. (2017). Frequent nonallelic gene conversion on the human lineage and its effect on the divergence of gene duplicates. Proceedings of the National Academy of Sciences of the United States of America, 114(48), 12779–12784. 10.1073/pnas.1708151114 29138319 PMC5715747

[humu24311-bib-0020] Harris, K. , & Nielsen, R. (2014). Error‐prone polymerase activity causes multinucleotide mutations in humans. Genome Research, 24(9), 1445–1454. 10.1101/gr.170696.113 25079859 PMC4158752

[humu24311-bib-0021] Hehir‐Kwa, J. Y. , Marschall, T. , Kloosterman, W. P. , Francioli, L. C. , Baaijens, J. A. , Dijkstra, L. J. , & Nijman, I. J. (2016). A high‐quality human reference panel reveals the complexity and distribution of genomic structural variants. Nature Communications, 7, 12989. 10.1038/ncomms12989 PMC5059695 27708267

[humu24311-bib-0022] Herberts, C. , & Wyatt, A. W. (2021). Technical and biological constraints on ctDNA‐based genotyping. Trends in Cancer, 7(11), 995–1009. 10.1016/j.trecan.2021.06.001 34219051

[humu24311-bib-0023] Kim , S. , Scheffler, K. , Halpern, A. L. , Bekritsky, M. A. , Noh, E. , Källberg, M. , Chen, X. , Kim, Y. , Beyter, D. , Krusche, P. , & Saunders, C. T. (2018). Strelka2: Fast and accurate calling of germline and somatic variants. Nature Methods, 15(8), 591–594. 10.1038/s41592-018-0051-x 30013048

[humu24311-bib-0024] Koboldt, D. C. , Zhang, Q. , Larson, D. E. , Shen, D. , McLellan, M. D. , Lin, L. , Miller, C. A. , Mardis, E. R. , Ding, L. , & Wilson, R. K. (2012). VarScan 2: Somatic mutation and copy number alteration discovery in cancer by exome sequencing. Genome Research, 22(3), 568–576. 10.1101/gr.129684.111 22300766 PMC3290792

[humu24311-bib-0025] Li, H. (2013). Aligning sequence reads, clone sequences and assembly contigs with BWA‐MEM. http://arxiv.org/abs/1303.3997

[humu24311-bib-0026] Li, H. , Handsaker, B. , Wysoker, A. , Fennell, T. , Ruan, J. , Homer, N. , Marth, G. , Abecasis, G. , Durbin, R. , & Genome Project Data Processing Subgroup (2009). The Sequence Alignment/Map format and SAMtools. Bioinformatics (Oxford, England), 25(16), 2078–2079. 10.1093/bioinformatics/btp352 PMC2723002 19505943

[humu24311-bib-0027] Li, W. , Freudenberg, J. , & Freudenberg, J. (2019). Alignment‐free approaches for predicting novel Nuclear Mitochondrial Segments (NUMTs) in the human genome. Gene, 691, 141–152. 10.1016/j.gene.2018.12.040 30630097

[humu24311-bib-0028] Lo Giudice, C. , Silvestris, D. A. , Roth, S. H. , Eisenberg, E. , Pesole, G. , Gallo, A. , & Picardi, E. (2020). Quantifying RNA editing in deep transcriptome datasets. Frontiers in Genetics, 11, 194. 10.3389/fgene.2020.00194 32211029 PMC7069340

[humu24311-bib-0029] Miga, K. H. , Koren, S. , Rhie, A. , Vollger, M. R. , Gershman, A. , Bzikadze, A. , Brooks, S. , Howe, E. , Porubsky, D. , Logsdon, G. A. , Schneider, V. A. , Potapova, T. , Wood, J. , Chow, W. , Armstrong, J. , Fredrickson, J. , Pak, E. , Tigyi, K. , Kremitzki, M. , … Phillippy, A. M. (2020). Telomere‐to‐telomere assembly of a complete human X chromosome. Nature, 585(7823), 79–84. 10.1038/s41586-020-2547-7 32663838 PMC7484160

[humu24311-bib-0030] Miller, T. L. A. , Orpinelli Rego, F. , Buzzo, J. L. L. , & Galante, P. A. F. (2021). sideRETRO: A pipeline for identifying somatic and polymorphic insertions of processed pseudogenes or retrocopies. Bioinformatics (Oxford, England), 37(3), 419–421. 10.1093/bioinformatics/btaa689 32717039

[humu24311-bib-0031] Naslavsky, M. S. , Scliar, M. O. , Yamamoto, G. L. , Wang, J. Y. T. , Zverinova, S. , Karp, T. , & Zatz, M. (2020). Whole‐genome sequencing of 1,171 elderly admixed individuals from the largest Latin American metropolis (São Paulo, Brazil). 10.1101/2020.09.15.298026

[humu24311-bib-0032] Penzkofer, T. , Jäger, M. , Figlerowicz, M. , Badge, R. , Mundlos, S. , Robinson, P. N. , & Zemojtel, T. (2017). L1Base 2: More retrotransposition‐active LINE‐1s, more mammalian genomes. Nucleic Acids Research, 45(D1), D68–D73. 10.1093/nar/gkw925 27924012 PMC5210629

[humu24311-bib-0033] Picelli, S. , Björklund, A. K. , Reinius, B. , Sagasser, S. , Winberg, G. , & Sandberg, R. (2014). Tn5 transposase and tagmentation procedures for massively scaled sequencing projects. Genome Research, 24(12), 2033–2040. 10.1101/gr.177881.114 25079858 PMC4248319

[humu24311-bib-0034] Plagnol, V. , Curtis, J. , Epstein, M. , Mok, K. Y. , Stebbings, E. , Grigoriadou, S. , Wood, N. W. , Hambleton, S. , Burns, S. O. , Thrasher, A. J. , Kumararatne, D. , Doffinger, R. , & Nejentsev, S. (2012). A robust model for read count data in exome sequencing experiments and implications for copy number variant calling. Bioinformatics (Oxford, England), 28(21), 2747–2754. 10.1093/bioinformatics/bts526 PMC3476336 22942019

[humu24311-bib-0035] Poplin, R. , Chang, P.‐C. , Alexander, D. , Schwartz, S. , Colthurst, T. , Ku, A. , Newburger, D. , Dijamco, J. , Nguyen, N. , Afshar, P. T. , Gross, S. S. , Dorfman, L. , McLean, C. Y. , & DePristo, M. A. (2018). A universal SNP and small‐indel variant caller using deep neural networks. Nature Biotechnology, 36(10), 983–987. 10.1038/nbt.4235 30247488

[humu24311-bib-0036] Poplin, R. , Ruano‐Rubio, V. , DePristo, M. A. , Fennell, T. J. , Carneiro, M. O. , Auwera, G. A. V. der , & Banks, E. (2018). Scaling accurate genetic variant discovery to tens of thousands of samples. BioRxiv , 201178. 10.1101/201178

[humu24311-bib-0037] Porubsky, D. , Garg, S. , Sanders, A. D. , Korbel, J. O. , Guryev, V. , Lansdorp, P. M. , & Marschall, T. (2017). Dense and accurate whole‐chromosome haplotyping of individual genomes. Nature Communications, 8(1), 1293. 10.1038/s41467-017-01389-4 PMC5670131 29101320

[humu24311-bib-0038] Rausch, T. , Zichner, T. , Schlattl, A. , Stutz, A. M. , Benes, V. , & Korbel, J. O. (2012). DELLY: Structural variant discovery by integrated paired‐end and split‐read analysis. Bioinformatics, 28(18), i333–i339. 10.1093/bioinformatics/bts378 22962449 PMC3436805

[humu24311-bib-0039] Swinnen, B. , Robberecht, W. , & Van Den Bosch, L. (2020). RNA toxicity in non‐coding repeat expansion disorders. The EMBO Journal, 39(1), e101112. 10.15252/embj.2018101112 31721251 PMC6939197

[humu24311-bib-0040] Thung, D. T. , de Ligt, J. , Vissers, L. E. , Steehouwer, M. , Kroon, M. , de Vries, P. , Slagboom, E. P. , Ye, K. , Veltman, J. A. , & Hehir‐Kwa, J. Y. (2014). Mobster: Accurate detection of mobile element insertions in next generation sequencing data. Genome Biology, 15(10), 488. 10.1186/s13059-014-0488-x 25348035 PMC4228151

[humu24311-bib-0041] Torene, R. I. , Galens, K. , Liu, S. , Arvai, K. , Borroto, C. , Scuffins, J. , Zhang, Z. , Friedman, B. , Sroka, H. , Heeley, J. , Beaver, E. , Clarke, L. , Neil, S. , Walia, J. , Hull, D. , Juusola, J. , & Retterer, K. (2020). Mobile element insertion detection in 89,874 clinical exomes. Genetics in Medicine, 22(5), 974–978. 10.1038/s41436-020-0749-x 31965078 PMC7200591

[humu24311-bib-0042] Tourdot, R. W. , Brunette, G. J. , Pinto, R. A. , & Zhang, C.‐Z. (2021). Determination of complete chromosomal haplotypes by bulk DNA sequencing. Genome Biology, 22(1), 139. 10.1186/s13059-021-02330-1 33957932 PMC8101039

[humu24311-bib-0043] van Heesch, S. , Mokry, M. , Boskova, V. , Junker, W. , Mehon, R. , Toonen, P. , de Bruijn, E. , Shull, J. D. , Aitman, T. J. , Cuppen, E. , & Guryev, V. (2013). Systematic biases in DNA copy number originate from isolation procedures. Genome Biology, 14(4), R33. 10.1186/gb-2013-14-4-r33 23618369 PMC4054094

[humu24311-bib-0044] Wenger, A. M. , Peluso, P. , Rowell, W. J. , Chang, P.‐C. , Hall, R. J. , Concepcion, G. T. , Ebler, J. , Fungtammasan, A. , Kolesnikov, A. , Olson, N. D. , Töpfer, A. , Alonge, M. , Mahmoud, M. , Qian, Y. , Chin, C. S. , Phillippy, A. M. , Schatz, M. C. , Myers, G. , DePristo, M. A. , … Hunkapiller, M. W. (2019). Accurate circular consensus long‐read sequencing improves variant detection and assembly of a human genome. Nature Biotechnology, 37(10), 1155–1162. 10.1038/s41587-019-0217-9 PMC6776680 31406327

[humu24311-bib-0045] Ye, K. , Guo, L. , Yang, X. , Lamijer, E.‐W. , Raine, K. , & Ning, Z. (2018). Split‐read indel and structural variant calling using PINDEL. Methods in Molecular Biology (Clifton, N.J.), 1833, 95–105. 10.1007/978-1-4939-8666-8_7 30039366

[humu24311-bib-0046] Zarate, S. , Carroll, A. , Mahmoud, M. , Krasheninina, O. , Jun, G. , Salerno, W. J. , Schatz, M. C. , Boerwinkle, E. , Gibbs, R. A. , & Sedlazeck, F. J. (2020). Parliament2: Accurate structural variant calling at scale. GigaScience, 9(12), giaa145. 10.1093/gigascience/giaa145 33347570 PMC7751401

[humu24311-bib-0047] Zhang, H. , Jain, C. , & Aluru, S. (2020). A comprehensive evaluation of long read error correction methods. BMC Genomics, 21(suppl 6), 889. 10.1186/s12864-020-07227-0 33349243 PMC7751105

